# Bioinformatics approach for the construction of multiple epitope vaccine against omicron variant of SARS-CoV-2

**DOI:** 10.1038/s41598-022-23550-w

**Published:** 2022-11-09

**Authors:** Sumera Zaib, Fatima Akram, Syed Talha Liaqat, Muhammad Zain Altaf, Imtiaz Khan, Ayed A. Dera, Jalal Uddin, Ajmal Khan, Ahmed Al-Harrasi

**Affiliations:** 1 Department of Basic and Applied Chemistry, Faculty of Science and Technology, University of Central Punjab, Lahore, 54590 Pakistan; 2 Department of Chemistry and Manchester Institute of Biotechnology, The University of Manchester, 131 Princess Street, Manchester, M1 7DN UK; 3 Department of Clinical Laboratory Sciences, College of Applied Medical Sciences, King Khalid University, Abha, Saudi Arabia; 4 Department of Pharmaceutical Chemistry, College of Pharmacy, King Khalid University, Abha, 62529 Kingdom of Saudi Arabia; 5 Natural and Medical Sciences Research Center, University of Nizwa, 616 Nizwa, Oman

**Keywords:** Biochemistry, Computational biology and bioinformatics

## Abstract

The World Health Organization categorized SARS-CoV-2 as a variant of concern, having numerous mutations in spike protein, which have been found to evade the effect of antibodies stimulated by the COVID-19 vaccine. The susceptibility to omicron variant by immunization-induced antibodies are direly required for risk evaluation. To avoid the risk of arising viral illness, the construction of a specific vaccine that triggers the production of targeted antibodies to combat infection remains highly imperative. The aim of the present study is to develop a particular vaccine exploiting bioinformatics approaches which can target B- and T-cells epitopes. Through this approach, novel epitopes of the S protein-SARS-CoV-2 were predicted for the development of a multiple epitope vaccine. Multiple epitopes were selected on the basis of toxicity, immunogenicity and antigenicity, and vaccine subunit was constructed having potential immunogenic properties. The epitopes were linked with 3 types of linker EAAAK, AAY and GPGPG for vaccine construction. Subsequently, vaccine structure was docked with the receptor and cloned in a pET-28a (+) vector. The constructed vaccine was ligated in pET-28a (+) vector in *E. coli* using the SnapGene tool for the expression study and a good immune response was observed. Several computational tools were used to predict and analyze the vaccine constructed by using spike protein sequence of omicrons. The current study identified a Multi-Epitope Vaccine (MEV) as a significant vaccine candidate that could potentially help the global world to combat SARS-CoV-2 infections.

## Introduction

Since the start of the pandemic, several variants were discovered along with the variants of concern (VOCs) that have major mutations with increased transmissibility, severity of the disease and resistance to vaccine^1^. The World Health Organization has designated VOCs as alpha, beta, delta, and gamma, containing mutations having a certain set of characteristics. Omicron was discovered in early November, 2021 in Botswana. On November 24, 2021, the World Health Organization reported the occurrence of omicron variant in South African countries and on November 26, 2021 it was on the list of variants of concern. An immense number of mutations recorded in omicron VOC in which the spike protein solely consists of 32 mutations, compared to the delta variant which was highly contagious but had only 16 mutations, as well as, NSP12 and NSP14, which are other viral replication proteins^2^.

Omicron variants have several assumptions postulated with respect to the probability of emerging patterns. For instance, (i) the potential for viral circulation among the chronically infected patients. (ii) At the time of the winter wave the introduction of the new variant in South Africa. (iii) The ability of spike to connect on host cell’s receptor ACE2 due to increased spike protein mutation. (iv) Animal reservoirs which are hidden may be the reason of very high rate of mutation in omicron variant. (v) The form of the omicron may also be due to low immunization rate of Africans in Africa^3^. Many concerns have been raised due to the emerging nature of omicron variant, including the source of exposure, the effect of mutations in response to vaccination, host immunity modification in response to mutations, lethality and potency of spreading omicron^4^.

The entry into the host cells is mediated by spike proteins that are highly glycosylated spike proteins of SARS-CoV-2. S. Perfusion trimer is thought to be destabilized by host cell’s angiotensin-converting enzyme 2 (ACE-2) receptor binding, which leads S1 subunit to shed and S2 subunit changing to helically elongated conformation, a pot-fusion change. Domains present at the top of the spike protein are most often targeted by neutralizing antibodies^5^.

Spike glycoprotein is the main unit where mutations in VOCs accumulate. In subunits of omicron named as RDB, NTD and S2, mutations are 15, 8 and 11, respectively. Out of these 34, six mutations were observed in other variants of interest or VOCs. Mutations included in RDB are E484A, Q493R, G496S, Q498R, N501Y, Y505H, G339D, S371L, S373P, S375F, K417N, N440K, G446S, S477N and T478K. Out of these mutations some known to have functional significance such as K417N, S447N, E484A, and Q493R, playing a specific role in immune escape. Moreover, higher infectivity is contributed by N501Y^6^. Spike protein mutations were 32 in the omicron form which were the largest number of mutations in mutant’s strains. Compared to the delta variant, there are twice the number of mutations in omicron. On the RBD of the omicron form, 10 mutations were found while in delta form only 2 mutations were reported. In alpha and delta variants, mutations were also found in spike proteins and their linkage was associated with increased transmissibility and ability to evade immune responses. Additionally, in omicron variant, N501Y mutation in spike protein has boosted its binding affinity with ACE-2 receptors facilitating the entry of the omicron variant into the host cells^7^. Furthermore, mutation in RBD at E484 in spike protein of omicron, circumvents human immune responses. Mutation also occurred at D614G, which might play a role in lungs epithelial cells with enhanced stability and transmission via viral replication. Furthermore, the alpha variant (B.1.1.7) and the mutation in R203K/G204R of the omicron variant in the N protein are related. By the virus evolution analysis, increased adaptability of R203K/G204R mutations would be observed using computation biology analysis. Several mutations in omicron were not previously reported, so their role in increasing severity of disease and infectivity still needs to be thoroughly studied and it would take time to explain characteristics of epidemiology, immune escape and transmissibility of omicron variant extensively^1^.

Un-vaccinated people are at more risk to get omicron variant. The plan of South African government was to vaccinate 300,000 people per day but the rate of vaccine administration is 120,000 people per day which was lower than the target. Most of the young patients who have moderate or severe COVID-19 had not been vaccinated or received only one shot. At the same time, 30–40% adults have been vaccinated in South Africa^8^. Good immunity can be provided by the use of vaccines against variants of SARS-CoV-2. Spike proteins are the main antigens on which most vaccines of SARS-CoV-2 vaccines are based for the activation of immune cells. So, evaluation of existing vaccines against omicron should be completed as soon as possible. To achieve acquired immunity against this disease, two to three shots of vaccines are necessary. If a person is exposed to SARS-CoV-2 or its variants, antibodies produced by vaccination against this infection would be sufficient to combat the disease^8^.

As the main component of the viral envelope, the S protein contributes to the assembly of viral particles with other cells and also plays role at the time of interaction and viral morphogenesis. Therefore, in this study, S-protein was targeted for designing multi-epitope vaccine of omicron variant using bioinformatics approaches that can be produced on an industrial scale in an appropriate host with suitable expression. Hence, the peptide was optimized in accordance to *E. coli*, as the most suitable and affordable expression system for the development of a vaccine. Ultimately, it was cloned in pET-28a ( +) vector, for expression analysis. The proposed/identified vaccine could be considered as a suitable therapeutic in the future after experimental validation (in vitro and in vivo testing).

## Methodology

The SARS-CoV-2 spike protein has a higher antigenic property, and its ability to enter and assemble viral genome makes it an auspicious target for developing vaccine construct. The S (spike) protein of coronavirus shows an important role in the process of cell membrane fusion and in receptor recognition. Furthermore, it is a suitable candidate for vaccine design due to its adhesive nature and low numbers of transmembrane helices, and there is no specific similarity of viral Spike protein with any protein of humans. In addition, it is also beneficial candidate due to its cellular localization^9^.

### Sequence retrieval and structure analysis of spike protein

NCBI (National Centre for Biotechnology Information Database) (https://www.ncbi.nlm.nih.gov/) was used for the selection of complete genomic sequence of SARS-CoV-2 with accession number NC_045512.29. The sequence of spike glycoprotein of SARS-CoV-2 was also retrieved from NCBI with accession number NC_045512.29^10^.

The physiochemical properties of the selected protein were checked by ExPASy-ProtParam tool (https://web.expasy.org/protparam/)^11^. ProtParam is a tool which allows the computation of various physical and chemical parameters for a protein that is stored in Swiss-Prot or TrEMBL or for a protein sequence entered by the user. The molecular weight, theoretical pI, amino acid and atomic composition, extinction coefficient, estimated half-life, instability index, aliphatic index, and grand average of hydropathicity (GRAVY) are among the parameters that were computed. The secondary structure of protein was analyzed by PSIPRED online tool (http://bioinf.cs.ucl.ac.uk/psipred/).The PSIPRED Protein Analysis Workbench is a web-based framework that brings together a number of different analysis tools. With access to GenTHREADER for protein fold recognition and MEMSAT-2 transmembrane topology prediction, it serves as an excellent tool for secondary structure prediction. Antigenicity of protein was checked using VaxiJen 2.0 (http://www.ddg-pharmfac.net/vaxiJen/VaxiJen/VaxiJen.html), the first server for alignment-independent prediction of protective antigens. Antigens can be classified using this server solely based on the physicochemical properties of proteins without recourse to sequence alignment^12^. Subsequently, allergenicity of protein was checked using AllerTOP v2.0, which is an online server (https://www.ddg-pharmfac.net/AllerTOP/method.html). AllerTOP is the first alignment-free server for in silico prediction of allergens based on the main physicochemical properties of proteins^13^. RaptorX is an online tool (http://raptorx.uchicago.edu/) used for the prediction of secondary structure and function of a protein. It is the most common method for protein structure prediction^14^. SWISS-MODEL (https://swissmodel.expasy.org/) is an online tool used for 3D (three-dimensional) modeling of a protein structure. It was used as a protein structure homology-modelling server, accessible via the Expasy web server, or from the program DeepView (Swiss Pdb-Viewer)^15^.

### Epitope prediction of B-cells using IEDB server

B-cell epitopes were predicted by using bioinformatics tool Immune Epitope Database (IEDB) (http://tools.iedb.org/main/). A complex algorithm is used in this tool that is based on protein antigen–antibody structure. It is an accurate, powerful, and high-quality software utilizing only epitope data from crystallized structures. IEDB is a free software and sponsored by NIAID. Experimental data was classified on the basis of human T-cell epitopes, autoimmunity, and infectious disease of other animal species, allergy, non-human primates and transplantation. This tool is also used for the analysis and prediction of epitopes^16^.

### T-cells epitopes prediction using IEDB server

The MHC-I binding prediction server was used in the IEDB tool for the prediction of conserved MHC-I binding epitopes. IEDB MHC-II binding predictions tool was used for the prediction of conserved MHC-II binding epitopes. Spike protein sequence was submitted in this server in FASTA format and SMM was used for prediction of MHC-I and MHC-II epitopes. Alleles were selected with a length of 9. The XHTML table was set as output format. The host species were selected as human and all additional options were selected as default. IEDB MHC-II binding prediction tool was used for the prediction of conserved MHC-II binding epitopes. SMM format was used for the prediction of MHC-II epitopes and sequence was submitted in FASTA format. All species of human HLA-DQ, HLA-DR, and HLA-DP were selected, and all alleles were set as default. XHTML table was set as output format and all additional options were selected as default^17^.

### B- and T-cell epitopes important features profiling

B- and T-cell epitopes antigenicity was analyzed using VaxiJen 2.0 tool. This server depends on physiochemical properties of proteins and it is an alignment-independent server^12^. The threshold value was set to 0.4 and the sequence was submitted in FASTA format, and all other parameters were set as default. AllerTop is an online tool that was used for allergenicity prediction of B- and T-cell epitopes^13^. Moreover, ToxinPred tool was used for the prediction of toxicity (http://crdd.osdd.net/raghava/toxinpred/)18.

### Population coverage calculation

The population coverage of the shortlisted epitopes was analyzed by using the IEDB’s Population Coverage (http://tools.iedb.org/population/) with default parameters. Different regions around the world were selected and the number of epitopes were also included. For the calculation of individuals that will respond in reaction to the epitopes set having known MHC restrictions, population coverage analysis tool was used. Following are the computations for the individual population coverage; 1) Expected population coverage, 2) Populations recognizing the average number of epitope hits/HLA combinations, 3) 90% of the population (PC90) recognizes the HLA combinations and the lowest number of epitope hits. HLA genotypic frequencies were used in these computations, under the assumption that there is no linkage disequilibrium between the HLA loci^19^.

### Conservancy analysis

For epitopes conservancy analysis of selected S protein, Immune Epitope Database (IEDB) was used and sequence of S protein was conserved^20^.

### Vaccine construction

Adjuvant sequence was taken from NCBI and all possible epitopes were used to develop multi-epitopes for the construction of vaccine. UniProt is a protein sequence and functional information database with many entries derived from genome sequencing projects. An adjuvant 50S ribosomal protein L3 (UniProtKB—P60438) (https://www.uniprot.org/) was used on N terminal of vaccine. EAAAK, CPGPG, and AAY are the types of linker used to construct vaccine sequence^21^.

### Assemblage of multi-epitope vaccine candidate sequence

To form a sequence of vaccine, B- and T-cell epitopes, adjuvants, CPGPG, AAY and EAAAK were arranged manually. To reduce the size of the vaccine construct, overlapping B-cell epitopes were combined. A merged sequence of epitopes produced a final vaccine^22^.

### Evaluation of the antigenicity and allergenicity of the vaccine construct

Vaccine antigenicity was predicted using an online server VaxiJen 2.0^12^, and allergenicity of multi-epitope vaccine was predicted using AllerTop^13^ while toxicity was predicted using an online server, ToxinPred, (it helps to predict and design toxic/non-toxic peptides and toxicity of peptides)^18^.

### Analysis of physiochemical properties of vaccine construct and its solubility

The physiochemical properties of a multi-epitope vaccine were predicted by ExPASy-ProtParam tool^11^. The protein-sol software was used to check the sequence of amino acid and required calculations were carried out for solubility prediction of multi-epitope vaccine. Solubility analysis was used to check the purity of a substance^23^.

### Secondary & tertiary structure extrapolation

The secondary structure of the constructed vaccine was predicted from RaptorX server^14^ and the 3D structure of the constructed vaccine using online server SWISS MODEL^15^. Both softwares predict main helix, coils and plates in the protein.

### Refinement of vaccine tertiary structure

The validation and refinement of the 3D structure of vaccine construct was carried out using an online server GalaxyRefine web server (https://galaxy.seoklab.org/cgi-bin/submit.cgi?type=REFINE), which is the most authentic tool for refinement and validation. In this method, repacking and side chains rebuilding were carried out. Subsequently, complete structure relaxation was done by molecular dynamics simulation procedure^24^.

### Vaccine tertiary structure validation

Three-dimensional (3D) structure was validated using RAMPAGE, Ramachandran Plot Assessment (https://zlab.umassmed.edu/bu/rama/). RAMPAGE is an offshoot of RAPPER which generates a Ramachandran plot. The results obtained from RAMPAGE were validated using PROCHECK (https://www.ebi.ac.uk/thornton-srv/software/PROCHECK/) which generates PostScript plots of a protein structure and residue-by-residue geometry for the examination of stereochemical quality. PROCHECK-NMR was included to verify the quality of NMR-solved structures^25^.

### Docking analysis

Molecular docking between the ligand binding domain of TLR3 receptor (PDB ID: 2A0Z) and the designed vaccine was carried out using an online server ClusPro 2.0 (https://cluspro.bu.edu/). The server requires two Protein Data Bank (PDB) files for basic use, however, it also provides a number of advanced search modification options. These include taking into account data from small-angle X-ray scattering (SAXS), building homo-multimers, applying attraction or repulsion, pairwise distance restraints, removing unstructured protein regions, and finding heparin-binding sites^26^.

### Molecular dynamics simulations

iModS (https://imods.iqfr.csic.es/) is an online server that is used for molecular dynamics simulations along with the prediction of torsional angles of the complex. Moreover, it explores the collective motions of proteins and nucleic acids using NMA in internal coordinates in addition to deformation of the structure, eigenvalue of interacting residues, RMSD values and co-variance among individual residues and stability of the complex were also analyzed in iModS^27^.

### Codon optimization of designed vaccine peptide for expression analysis

To express the chimeric protein in an expression system, the amino acid sequence was reverse translated by backtranseq program of EMBOSS 6.0.1 (https://www.ebi.ac.uk/Tools/st/emboss_backtranseq/). EMBOSS software analysis package automatically copes with data in a variety of formats and allows transparent retrieval of sequence data from the web^28^, followed by codon optimization using Java Codon Adaptation tool (JCat) (http://www.jcat.de/), an online web-based server. It helps in the adjustment of codon usage of an input sequence to the selected organism, which is useful for improving the expression of foreign genes in hosts with different codon usage^29^. This was done to express the chimeric peptide in *Escherichia coli* (*E. coli*-K12 strain), because a few codons in *E. coli* encode different amino acids compared to the native host. To perform codon optimization, the nucleotide sequence of construct was pasted and selection was made in order to avoid rho-independent transcription termination, prokaryotic ribosome binding, and restoration of enzyme cleavage sites. Subsequently, the results were obtained in the form of GC content percentage and codon adaption index (CAI) before and after optimization of the input (query) sequence. The acceptable CAI score of optimized sequence lies between 0.8–1.0, where 1.0 is scrutinized as an ideal score. Additionally, GC content should be between 30–70% for optimized sequence as the transcriptional and translational performance will be affected by the value outside this range. Furthermore, *E. coli* pET-28 ( +) was selected as an expression vector for vaccine construct from SnapGene plasmid library (https://www.snapgene.com/resources/plasmid-files/). SnapGene helped in planning, observing, and documenting DNA cloning, PCR and primer design. Subsequently, vaccine construct and *E. coli* pET-28 ( +) were analyzed for restriction sites via SnapGene tool. SgrAI and NgoMIV restriction sites were used to produce flanking sites at the ends of the construct and plasmid that make the expression of the vaccine construct feasible. In silico polymerase chain reaction (PCR) was also performed to amplify the construct using SnapGene^30^.

## Results

### Sequence and structure analysis

The SARS-CoV-2 S protein sequences were extracted from the ViPR database for developing a vaccine candidate. As part of the receptor recognition and fusion process of the cell membrane, the spike protein (S) of SARS-CoV-2 has two subunits, S1 and S2. One of the most virulent viral proteins, S, was discovered to be antigenic. Using VaxiJen 2.0, an internet server, we were able to estimate the antigenicity of viral protein^12^. To increase specificity, the cutoff point was lowered from 0.4 to 0.425. An examination of the full-length protein antigenicity demonstrated that S protein has a 0.4451 antigenicity making it a potential antigen. This protein was then further investigated. ProtParam^11^ was used to calculate the physiochemical properties of SARS CoV-2 S protein which showed a molecular weight of 1,441,178.47 Da whereas it contains 1273 amino acid units. According to our calculations, the theoretical isoelectric point (pI) was found to be 6.24, which indicates that the protein has a positive charge as beyond this point protein shows negative charge. Our protein was classified as stable by ProtParam because of its low instability index (II) of 33.01. The protein was found to be thermostable bearing a wide range of temperatures according to its aliphatic index of 84.67. The formula used for the detection of the total number of carbon (C), hydrogen (H), nitrogen (N), oxygen (O) and sulfur (S) was C_6336_H_9770_N_1656_O_1894_S_54_.

### Prediction of 2D and 3D structures

**Figure 1:**
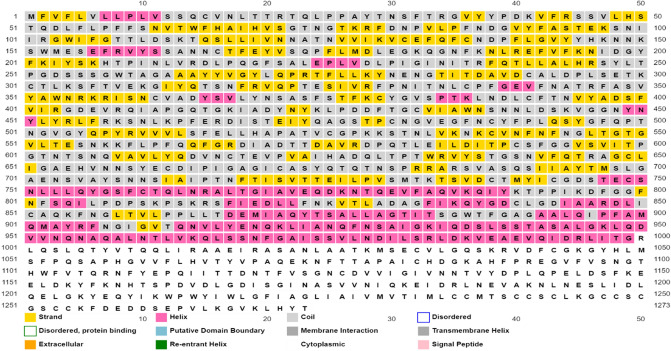
Prediction of secondary structure via PSIPRED^31^.

PSIPRED is an online server used to predict secondary structure^31^ and 3D structure was predicted using an online server SWISS-MODEL^15^. RaptorX showed that spike protein revealed 30% loops, 10% *β* sheets and 10% *α* helixes as shown in Fig. 1.

The total length of residues was 1273, the number of predicted TMH was 1, the exported number of AAs in TMH was 23.97303, and the total prob of N terminal was 0.00077. Moreover, the residues from 1 to 1273 were found in transmembrane region, residues from 1 to 1213 were surface exposed and 1214–1236 residues were found inside the TM helix of spike protein as shown in Fig. S1^32^.

### Identification of B-cell epitopes

**Figure 2:**
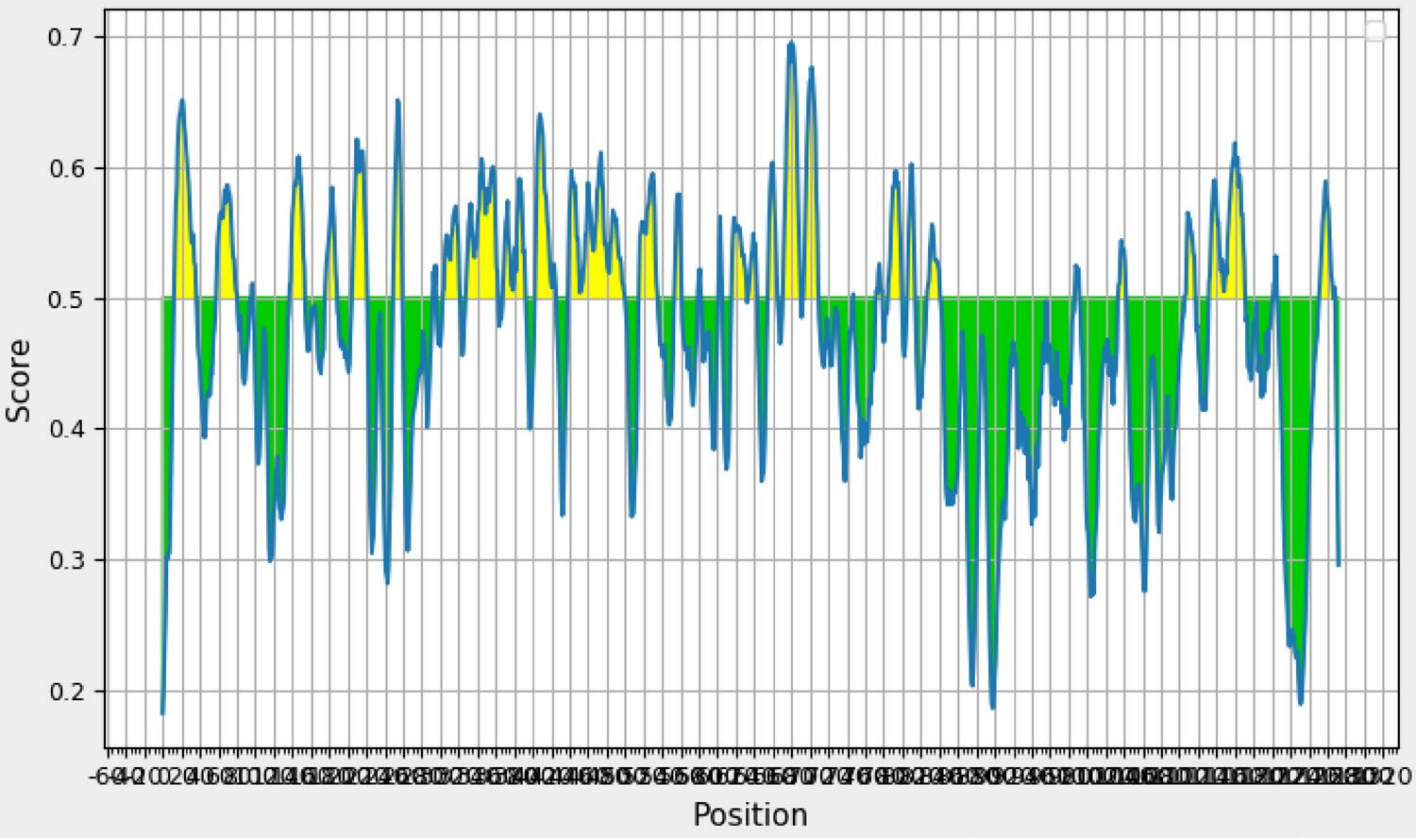
BepiPred linear epitopes predicted via IEBD analysis resource^20^.

For resistance to viral infection, B-cell epitopes play an important role. There are different features of potential B-cell epitopes that include recognizing B-cells along with the unlikely immune responses activation beside typical infection of virus. For B-cell epitope prediction, amino acid screened based method was used. The BepiPred linear epitope prediction method was used that predicted 40 linear epitopes with 0.350 threshold score. The linear epitopes prediction maximum score was 2.291 while minimum at − 0.001. Additionally, the average score predicted by BepiPred linear epitopes prediction was − 0.066. The peptide sequences from 181–186 amino acids declared as B-cell epitopes and can speed up the preferred immune response due to its highest antigenicity value 2.1342, non-allergenicity and non-toxicity as shown in Fig. 2 and Table 1.

**Table 1:** BepiPred linear epitopes prediction using IEBD server^20^.

No	Start	End	Peptide	Length	Antigenicity	Allergenicity	Toxicity
1	71	81	SGTNGTKRFDN	11	0.5906	Allergen	Toxic
2	181	186	GKQGNF	6	2.1342	Non-allergen	Non-toxic
3	282	287	NGTITD	6	1.1184	Allergen	Toxic
4	318	324	FRVQPTE	7	1.6729	Allergen	Toxic
5	407	420	VRQIAPGQTGKIAD	14	1.2606	Non-allergen	Non-toxic
6	439	447	NNLDSKVGG	9	0.8904	Allergen	Toxic
7	567	580	RDIADTTDAVRDPQ	14	0.4400	Allergen	Toxic
8	805	816	ILPDPSKPSKRS	12	0.5322	Non-allergen	Non-toxic
9	936	941	DSLSST	6	0.7417	Allergen	Toxic
10	1157	1167	KNHTSPDVDLG	11	1.4039	Non-allergen	Non-toxic

**Figure 3:**
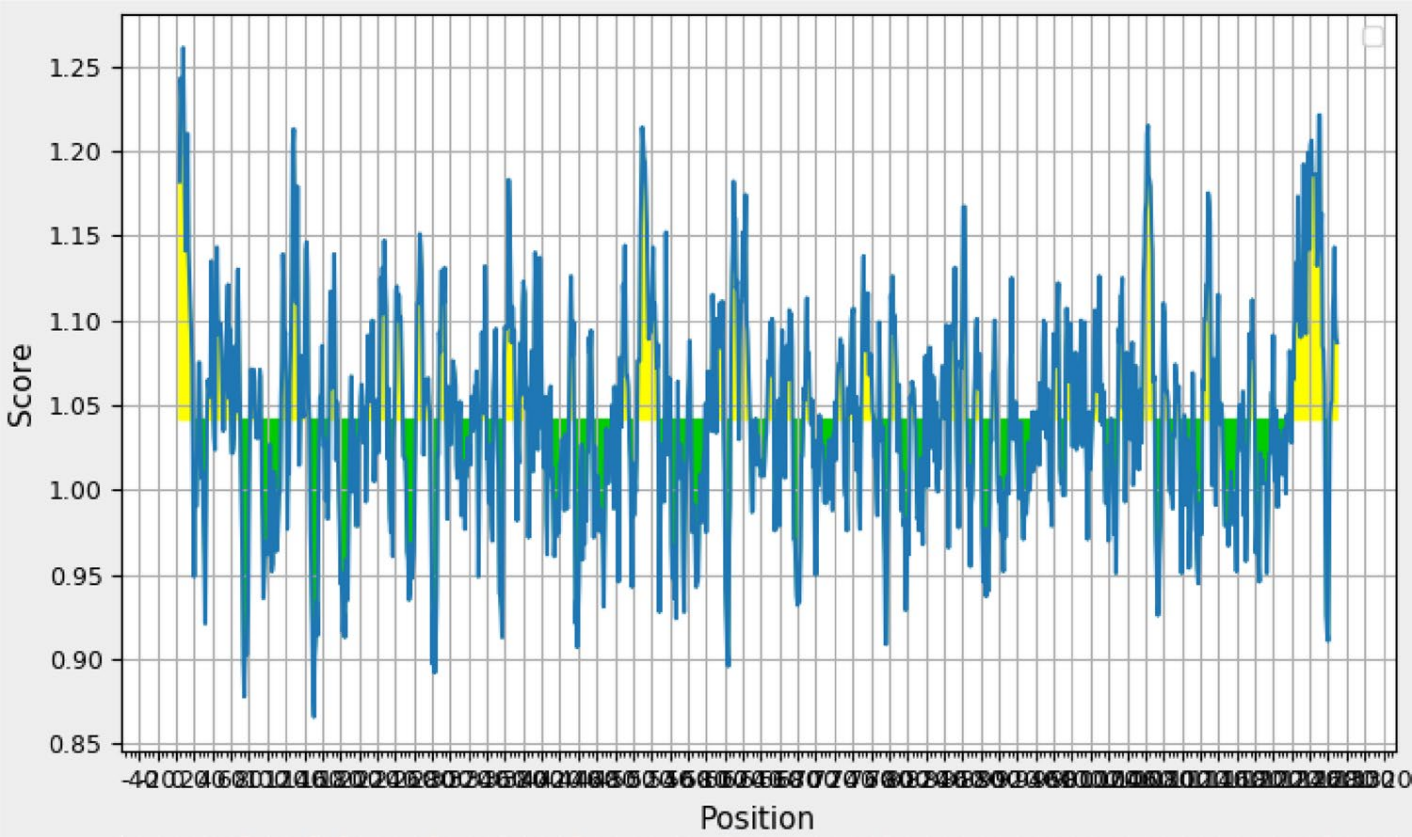
Antigenicity analysis by Kolaskar and Tongaonkar method^33^.

Another method from Kolaskar and Tongaonkar was used for the prediction of known amino acid antigenicity^33^. The antigenicity maximum propensity was 1.1443 while the minimum tendency of antigenicity was − 1.3917. Threshold value greater than 1.00 was candidate of antigenic dynamic and the value was set as 1.00. For further analysis, 13 B-cell epitopes were separated based on threshold value, antigenicity and allergenicity. Sequences from 288 to 295 and 847–853 have ability to initiate the B-cell response (Fig. 3 and Table 2).

**Table 2:** List of known amino acid antigenicity predicted using Kolaskar and Tongaonkar method^33^.

No	Start	End	Peptide	Length	Antigenicity	Allergenicity	Toxicity
1	34	41	RGVYYPDK	8	1.0191	Allergen	Toxic
2	44	51	RSSVLHST	8	0.5495	Allergen	Toxic
3	210	216	INLVRDL	7	− 0.3198	Allergen	Toxic
4	223	230	LEPLVDLP	8	− 0.3271	Allergen	Toxic
5	272	278	PRTFLLK	7	− 1.3917	Allergen	Toxic
6	288	295	AVDCALDP	8	0.773	Non-allergen	Non-toxic
7	617	627	CTEVPVAIHAD	11	0.0499	Allergen	Toxic
8	803	808	SQILPD	6	− 0.1542	Allergen	Toxic
9	837	843	YGDCLGD	7	− 0.5555	Allergen	Toxic
10	847	853	RDLICAQ	7	1.1443	Non-allergen	Non-toxic
11	973	979	ISSVLND	7	0.0414	Allergen	Toxic
12	1030	1037	SECVLGQS	8	− 0.0110	Allergen	Toxic
13	1079	1085	PAICHDG	7	− 1.0100	Allergen	Toxic

**Figure 4:**
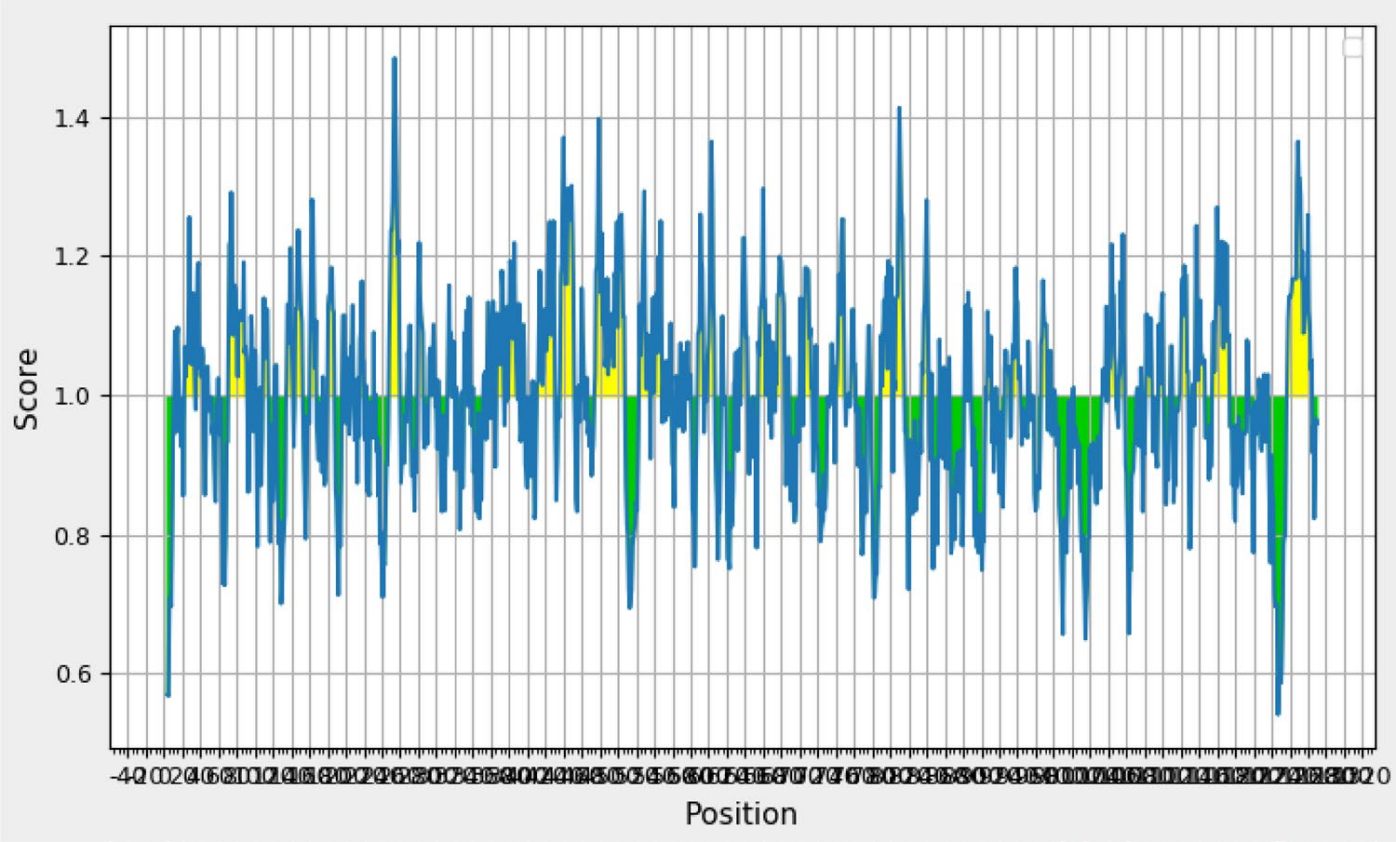
Good surface accessibility by Chou-Fasman method^35^.

Good surface accessibility was predicted using Emini surface accessibility tool^34^ and it has been very effective factor for B-cell epitopes prediction. 22 Epitopes were separated by passing default threshold value. Average score predicted by Emini surface accessibility tool was 1.00, maximum value was 1.8367, while − 1.0253 was the minimum. Out of 22 epitopes, 6 were further screened out on the basis of antigenicity and allergenicity (Fig. 4 and Table 3).

**Table 3:** List of good surface accessibility peptides^34^.

No	Start	End	Peptide	Length	Antigenicity	Allergenicity	Toxicity
1	73	80	TNGTKRFD	8	0.2041	Allergen	Toxic
2	110	115	LDSKTQ	6	1.3071	Allergen	Toxic
3	179	185	LEGKQGN	7	1.8367	Non-allergen	Non-toxic
4	202	208	KIYSKHT	7	0.7773	Non-allergen	Non-toxic
5	250	255	TPGDSS	6	0.3268	Allergen	Toxic
6	278	284	KYNENGT	7	0.9414	Allergen	Toxic
7	352	357	AWNRKR	6	1.6320	Non-allergen	Non-toxic
8	419	428	ADYNYKLPDD	10	0.6956	Allergen	Toxic
9	437	442	NSNNLD	6	1.1859	Non-allergen	Non-toxic
10	455	468	LFRKSNLKPFERDI	14	0.3610	Allergen	Toxic
11	569	581	IADTTDAVRDPQT	13	0.3018	Allergen	Toxic
12	601	606	GTNTSN	6	1.2831	Non-allergen	Non-toxic
13	674	685	YQTQTNSPRRAR	12	0.0760	Non-allergen	Toxic
14	773	779	EQDKNTQ	7	0.1017	Allergen	Toxic
15	786	794	KQIYKTPPI	9	0.2705	Allergen	Toxic
16	808	817	DPSKPSKRSF	10	0.8148	Non-allergen	Toxic
17	1068	1076	VPAQEKNFT	9	1.0107	Non-allergen	Non-toxic
18	1105	1111	TQRNFYE	7	0.2190	Non-allergen	Toxic
19	1139	1162	DPLQPELDSFKEELDKYFKNHTSP	24	− 0.3378	Non-allergen	Toxic
20	1179	1186	IQKEIDRL	8	− 1.0253	Allergen	Toxic
21	1202	1210	ELGKYEQYI	9	0.5415	Allergen	Toxic
22	1256	1261	FDEDDS	6	− 0.0685	Allergen	Toxic

Beta turn plays a vital role in the beginning of immune defense response and beta turn is hydrophilic in nature and exposed to the surface. Spike protein beta turn was predicted using Chou- Fasman beta turn evaluating algorithm^35^. Default threshold value was adjusted at 0.997. The average score predicted by Chou-Fasman was 0.997, whereas, 0.541 and 1.484 were minimum and maximum scores, respectively. Chou and Fasman graphical representation is shown in Fig. 4. Several studies have suggested that the antigenicity of protein is linked with flexibility of peptides. To test this, Karplus-Schulz method was used^36^. Threshold value was adjusted to 0.993. The maximum score predicted by Karplus-Schulz method was 1.125, the minimum score was 0.876 and the average score was 0.993. The graphical representation of Karplus and Schulz is shown in Fig. 3. Further epitopes were screened on the basis of toxicity, antigenicity and allergenicity. Non-allergic, non-toxic and antigenic epitopes were chosen and toxic, allergic and non-antigenic epitopes were removed. Furthermore, conservancy analysis was performed using an online IEBD conservancy analysis tool^20^. Total 12 B-cell epitopes were used and selected in vaccine construction at 100% coverage and identity.

### Prediction of T-cell epitopes

#### Prediction of MHC-II binding profile

The epitopes of MHC-I T cells were predicted using an online server IEDB. MHC-I T-cell epitopes HLA binding affinity was also predicted by this tool and these predictions were based on IC_50_ nM unit. A lower IC_50_ value shows a higher binding affinity of the MHC-I T cell epitopes. The IEDB tool predicted a total of 2024 MHC-I T-cell epitopes. Further epitopes were screened out. These epitopes were based on IC_50_ less than 200.458 MHC-I T-cell epitopes were separated on the basis of allergenicity, toxicity and antigenicity. 150 MHC-I T-cell epitopes were concluded for vaccine construction. The main epitope TKLNDLCFT showed highest antigenic score of 2.9364.

#### Prediction of MHC-II binding profile

IEDB tool predicted a total of 3475 MHC-II T-cell epitopes. Of these, 699 MHC-II T-cell epitopes were designated based on the basis of MHC-II alleles and epitope interaction. At last, 179 epitopes out of 699 were separated on the basis of their toxicity, antigenicity and allergenicity. The main epitope QIAPGQTGKIADYNY showed a maximum antigenic score of 1.6088.

#### Vaccine construction

A total of 12 B-cell epitopes were taken for vaccine construction. 50S ribosomal protein L3 with UniProtKB-P60438 was used for vaccine construction as an adjuvant. Three types of linkers were used to build vaccine. For the generation of specific immune response, a linker EAAAK was used that links B-cell epitopes and adjuvant at the amino N-terminal. Furthermore, T-cells and linkage of B-cells was made possible with the help of CPGPG and AYY linker, as shown in Fig. S2. At the C amino terminal of vaccine sequence, 6 × histidine was fused for protein purification and identification process. The final constructed sequence showed theoretical pI = 8.33, amino acid = 1716 and molecular weight of 189,005.43.

#### Vaccine protein antigenicity and allergenicity assessment

Vaccine protein antigenicity with adjuvant and non-adjuvant was predicted using an online server VaxiJen 2.0 server^12^. Antigenicity with adjuvant was 0.7997 while the non-adjuvant value was 0.8375. The nature of constructed vaccine is antigenic whether it is linked with adjuvant or not. Constructed vaccine toxicity was predicted by Toxinpred^18^ and found to be non-toxic.

#### Analysis of solubility and physicochemical properties of multi-epitope vaccine

**Figure 5:**
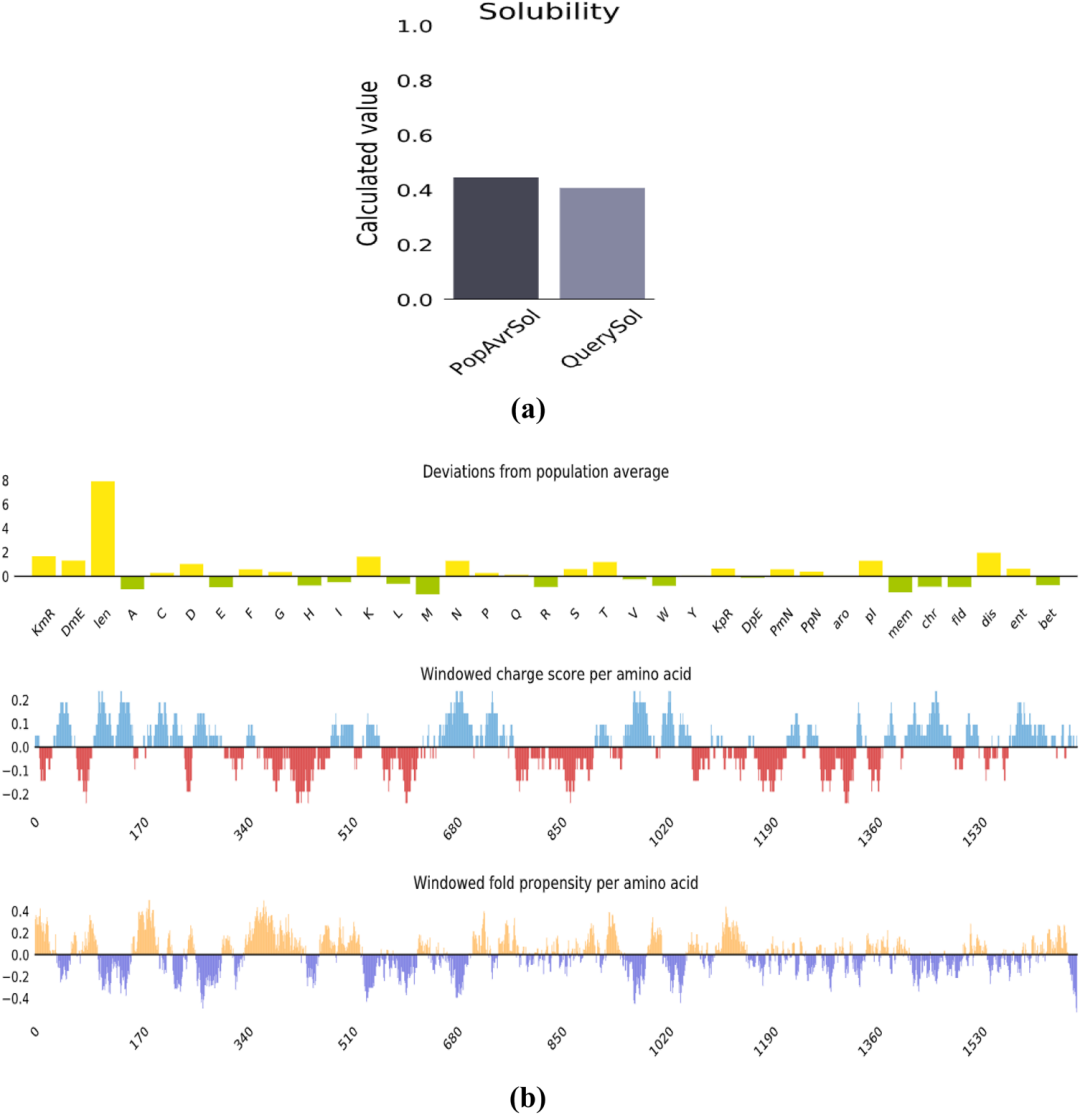
(**a**) Solubility rate of multi-epitopes vaccine calculated through Protein-Sol server. (**b**) Deviation from population average and charge score of multi-epitopes vaccine calculated through Protein-Sol server^23^.

ExPASY ProtParam tool^11^ was used to predict physicochemical properties and protein nature. The molecular weight of multi-epitope vaccine was 189,005.43 Da. Protein calculated pI was 8.38, revealing the basic nature of protein. The protein instability index (II) was 29.36. Instability index calculation indicated that a protein is stable if it shows a value > 40. Aliphatic index values were found to be 76.19 and GRAVY index was − 0.523 indicating a stable protein. GRAVY negative value indicated the protein to be hydrophilic in nature. Our vaccine construct showed good solubility rate calculated through Protein-Sol server with the score of 0.408 (Fig. 5a,b).

#### Prediction of secondary structure of vaccine

RaptorX^14^ is an online server used to analyze the nature of protein and to predict secondary structure. The secondary structure prediction results showed that the protein contains 26% beta strands, 68% coils and 7% helix. 24% Protein was present in exposed region, 23% in medium exposed and 3% of the protein residues were present in disordered region (Table 4).

**Table 4:** Secondary structure prediction of multi-epitopes vaccine using RaptorX^14^.

Secondary structure prediction			
Secondary structure	7%H	24%E	68%C
Solvent access	50%E	23%M	26%B
Disordered	3%		

#### Prediction of protein tertiary structure

**Figure 6:**
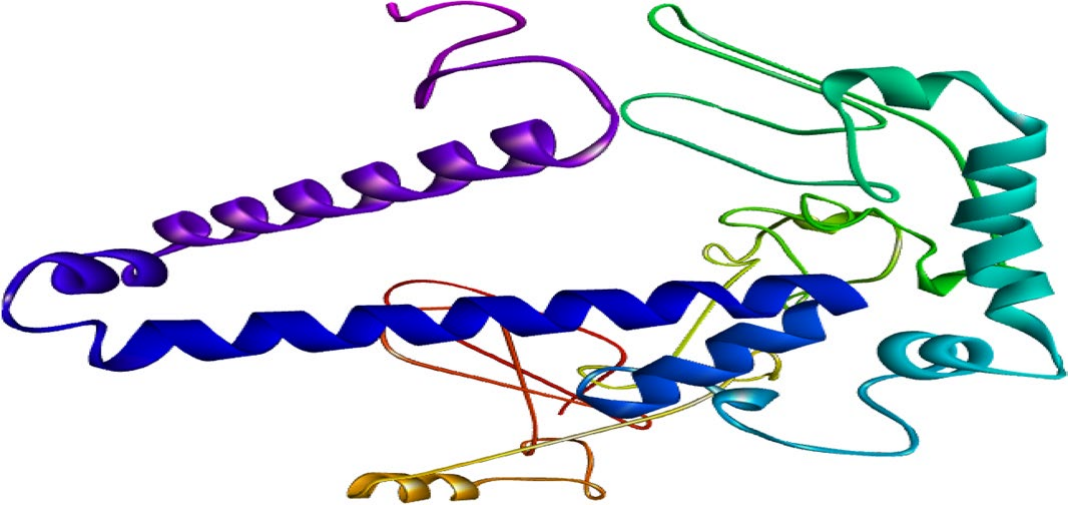
3D vaccine structure using SWISS-MODEL^15^.

SWISS-MODEL^15^ is an online server used to construct the best 3D structure of a chimeric vaccine. All the models were analyzed and are based on high coverage values. The model with best coverage score was used for refinement method (Fig. 6).

#### Tertiary structure refinement using GalaxyRefine

GalaxyRefine tool^24^ was used to refine the vaccine construct. This tool also provided 5 models of the vaccine. There are some parameters involved in refinement method, like MolProbity (2.079), GDT-HA (0.9581) and RMSD (0.388). The clash score predicted by GalaxyRefine was 9.2, Ramachandran score was 88.4% and poor rotamers score was 0.6 (Table 5). Model 3 was the best refined vaccine structure and was further used for simulation.

**Table 5:** Refine models value for multi-epitopes vaccine using GalaxyRefine^24^.

Model	GDT-HA	RMSD	MolProbity	Clash score	Poor rotamers	Rama favored
Initial	1.0000	0.000	3.105	8.5	13.4	75.4
MODEL 1	0.9749	0.358	2.138	10.7	0.6	88.4
MODEL 2	0.9617	0.372	2.052	8.6	0.6	88.4
MODEL 3	0.9581	0.388	2.079	9.2	0.6	88.4
MODEL 4	0.9605	0.373	2.167	12.3	0.6	89.4
MODEL 5	0.9629	0.367	2.104	10.1	0.0	88.9

#### Validation of 3D structure of designed vaccine

RAMPAGE is an online server^25^ used to validate and analyze the refined structure of vaccine. For 3D structure validation of designed vaccine, Ramachandran plot was used (Fig. S3). In Ramachandran plot, 93% region is present in favored region, 0.7% structure was in disallowed region and 5.7% structure was in allowed region.

#### Molecular modeling of vaccine construct with ligand binding domain of ACE2

**Figure 7:**
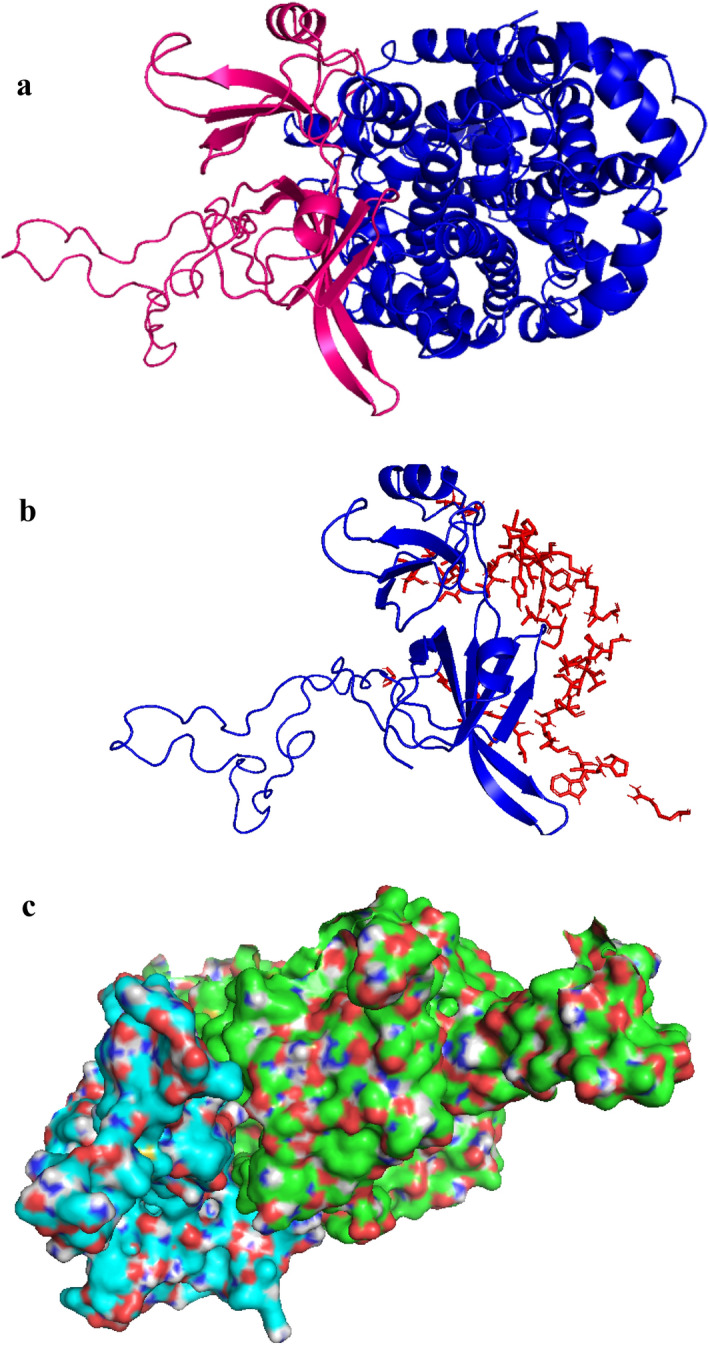
Best docked model of multi-epitope vaccine/receptor using (**a**) ClusPro^26^, (**b**) PyMOL^37^ and (**c**) cartoon model.

ClusPro2.0^26^ is an online server used for molecular docking of proteins and it was used for the estimation of refined vaccine model interaction by ligand binding domain of immune receptor ACE2. Multiple models were provided by docking studies. After checking 29 docked poses of the complex, top model was selected on the basis of active sites, having center energy − 1035.4 kJ/mol and lowest energy − 1215.1 kcal/mol, using PyMOL^37^ (Fig. 7a,b,c).

#### Molecular dynamics simulations

**Figure 8:**
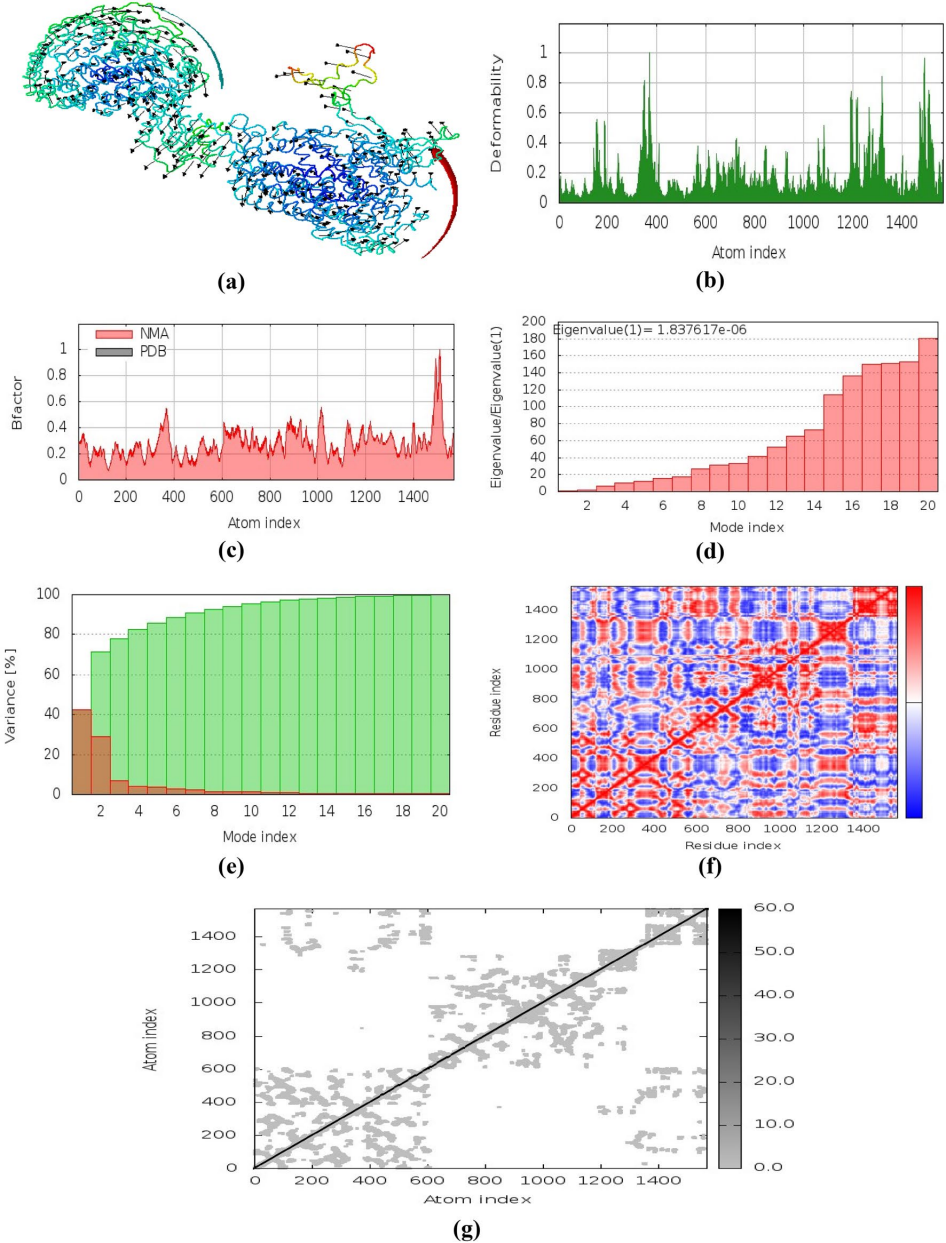
Molecular dynamics simulations of docked complex. (**a**) MNA mobility of protein structure, (**b**) deformability of complex, (**c**) B-factor, (**d**) Eigen value, (**e**) cumulative variance shown in green color and variance in red color, (**f**) correlated, anti-correlated and noncorrelated variance map indicated by red, blue and white color, respectively, and (**g**) shows elastic network (stiffer regions indicated by darker grey color)^27^.

iMODS is an online server^27^ that provides a critical structure study by adjusting a complex force field with different time intervals. Less deformation present at every residue. The complex eigenvalue is 1.837617e-06. The better interaction of every residue was revealed due to low RMSD and highly correlated areas in heat maps (Fig. 8). This figure shows (a) protein structure with its MNA mobility, (b) low deformability present in every residue, (c) B-factor, (d) Eigen value 1.837617e-06, (e) variance in both green and red color, (f) and (g) indicated the elastic network and covariance of the complex.

#### Codon optimization of designed vaccine peptide for expression analysis

**Figure 9:**
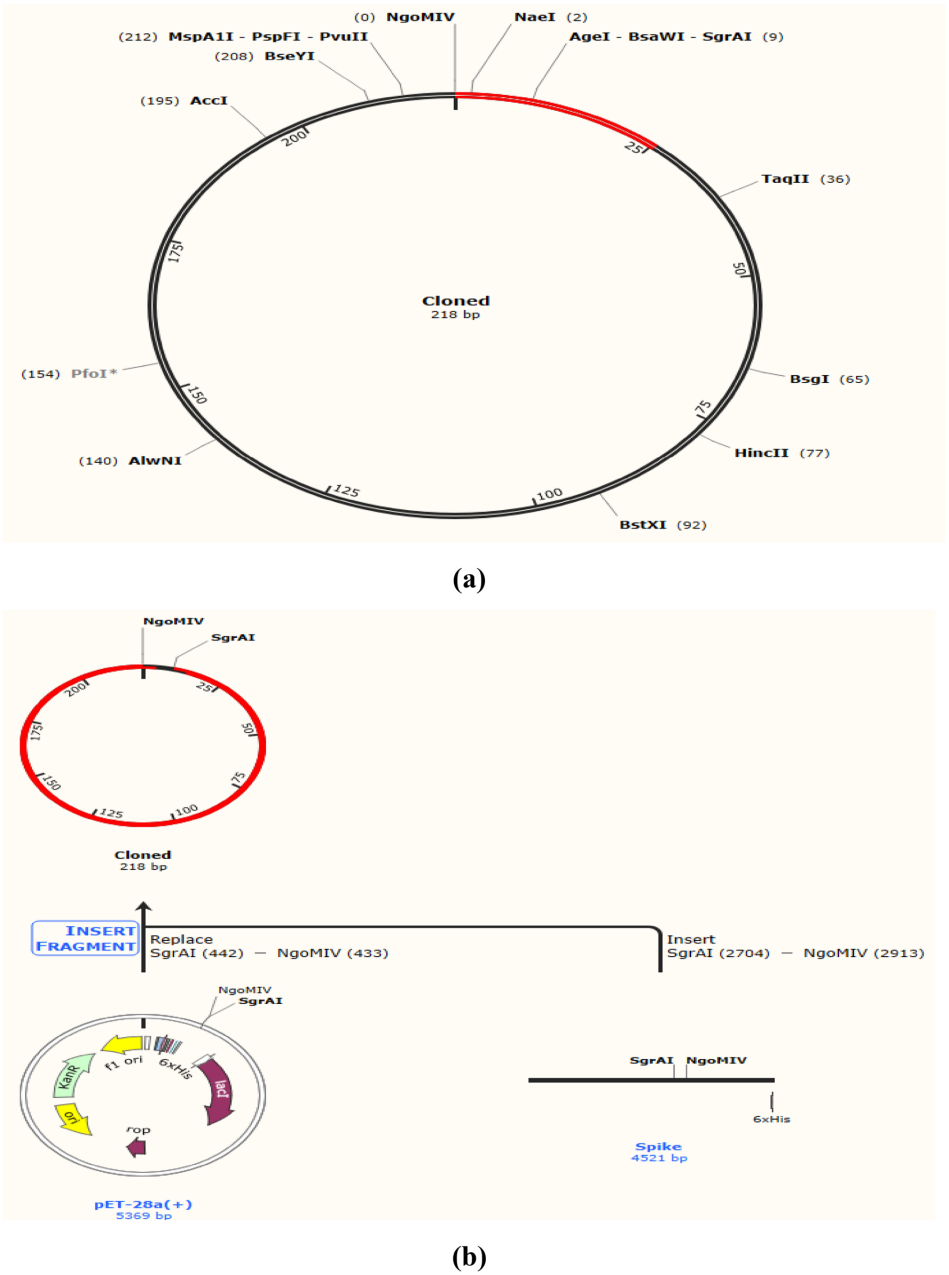
(**a**) The optimized sequence was cloned in pET-28a (+) vector to make a recombinant plasmid after being amplified through in silico PCR by using SnapGene software^30^. (**b**) In silico PCR amplification of vaccine construct followed by addition of restriction sites and cloning in pET-28a ( +) vector.

In the in silico cloning process the crucial step is expression system of *E. coli* for an efficient vaccine expression. Linkers and adjuvants suitable with prioritized B- and T-cell epitopes, were used to design vaccines. In the Java codon adaptation tool (JCat) as input (individually), nucleotide sequence of these vaccines was used for adaptation of codon to the prokaryotic organisms which are mostly sequenced. Codon optimization was carried out using the Java codon adaptation tool (JCat)^29^, which allowed the highest possible levels of protein production. According to the CAI for the optimized codon, which had a length of 927 nucleotides and a GC content of 50.52%, the sequence before and after adaptation was both shown to have a CAI of 1. Both sequences are presented in Fig. S4a, b. *E. coli* GC concentration ranges from 30 to 70%, which is regarded as optimal for steady vector expression in *E. coli.* Using SnapGene software^30^, the optimized sequence was cloned into the pET-28a (+) vector which created a new plasmid following in silico PCR, making it a recombinant plasmid (Fig. 9a,b).

## Discussion

Following an increase in global crisis due to some deadly viruses, it is essential to advance in finding an appropriate cure for the diseases. For designing a peptide-based vaccine bioinformatics tools represent a vital approach. For targeting a viral disease, the peptide-based vaccine has proven to be a promising strategy as seen in dengue, chikungunya, SLE and rhinoviruses^38^. However, in RNA-based vaccine the resistance to virus is due to the high rate of mutation in the SARS-CoV-2 genome. Our study focuses on spike protein. The antigenicity of the helical target protein was determined to be an excellent vaccination candidate. Bioinformatics software was used to create the PDB structure of the linked protein, which was previously unavailable in other protein data banks. Overall, in silico approach is being used in our study elaborating the multi-epitope-based design of the vaccine. This study helps to boost the immune response for cell-mediated as well as humoral reactions.

A study reported the reverse vaccinology method by predicting MHC I and MHC II classes and compared mutational analysis between BA.1 and BA.2 for vaccine construct ^39^. B-cells were used only in the past for the potential vaccine design and source. This study targeted MHC-B and MHC-T classes with human leukocyte antigen (HLA). A period of antigenic drift may eventually free the antigen from its memory-induced antibody response. Although T-cell immunity provides a long-lasting immune response, the process of epitope-to-vaccination includes certain rigorous conditions. We began by creating a database of all conceivable S protein epitopes. Antigenicity predictions for B-cell epitopes were made using five IEDB database approaches. Different types of parameters including flexibility, hydrophilicity, peptide chains and their antigen propensity, surface polarity, and accessibility are all related to epitope position. The results of the Kolaskar and Tongaonkar^33^, Chou and Fasman^35^, Karplus and Schulz^36^, Emini surface^34^, and BepiPred linear epitope calculations provide a visual representation of each residue's potential role in epitope generation. There are several T-cell epitopes with IC_50_ values less than 200 that are very active against their targeted allele. Using the consensus technique, epitopes that interact with more than five MHC Class-I and Class-II molecules are isolated for further testing.

For both B- and T-cells, the antigen property was checked primarily. ACC calculation was done using the VaxiJen v2.0^12^, and these were performed on the basis of their physicochemical properties. Peptides with a value greater than 0.4 found to be good. To elicit an effective immune response, the antigenic epitope must be nontoxic in nature. For predicting the toxicity of peptides, SVM classifier were used by ToxinPred^18^.

Another study discovered epitopes conservity targeting SARS-CoV-2 mutations in different variants. The conservity was checked by predicting epitopes with the help of immune epitope database (IEDB). This method provides a scientific approach for selecting epitopes with good immunogenicity and conservity against any virus^40^. Currently, immune system stimulation triggers allergic reaction in response to the vaccines developed. Based on the quantitative structure–activity relationship (QSAR), AllerTop v.2.0^13^ gives us the score value which makes allergen probability. An epitope filtered out by the IEDB has a high degree of conserved protein sequences and similarity among strains. Furthermore, IEDB was used for the T-cell analysis of population coverage as polymorphic property is found in MHC molecules. This is also found in alleles of human MHC (HLA) which is diverse in nature. There is a high level of conservation in both classes of MHC over the world. After meeting all the requirements, 12 B-cell epitopes, 179 MHC Class-II T-cell epitopes and 150 MHC Class-I T-cell epitopes were selected as vaccine components.

An increased response of the vaccine against the recognition of the pathogen was observed through the involvement of the 50S ribosomal protein L3 in addition to activation of the immune system^41^. Thus, this was used as an adjuvant which increased the immune-reactive property. In order to create the multi-epitope subunit vaccine, chosen B- and T-cell epitopes were first linked together using appropriate linkers. Spacer sequences are crucial to the creation of vaccines because of their ability to maximize efficacy. GPGPG and AAY linkers were included between the anticipated epitopes in earlier experiments to generate a vaccine with the best antigenicity possible. In the sequence designed, an EAAAK linker is adjusted which attaches the adjuvant to the first predicted B-cell epitope. Fusion proteins can be enhanced by the entanglement of this linker which has been documented. At the C terminal of sequence, there is a 6xHis-tag, also known as poly-histidine tag, consisting of 6 histidine residues which is attached to carboxyl (C-) terminal. When the histidine residues are bound to the immobilized ions, they make it easier for the sequence to function in the buffer conditions.

Using bioinformatics and immunology, researchers have found that the created protein sequence do not possess any allergenic or poisonous qualities. Vaccine antigenicity has been shown to be of little utility in a few recorded investigations. However, the designed chimeric vaccine had an acceptable antigenic score regardless of whether it was coupled with adjuvant or not. The proposed vaccine protein's molecular weight was determined to be 189,005.43 Da, and its solubility was examined in light of its induced antigenicity. In accordance with the vaccine protein and its basic nature, the pI value was 8.38. When having a value of 29.36, there will be an instability index shown by the vaccine. Therefore, it is possible to use this protein as a vaccination model after successful in vitro and in vivo testing. According to the calculation of aliphatic index, the construct of the chimeric vaccine was developed to be thermostable.

For the construction of a vaccine, the tertiary and secondary structures are very essential. About 7% of the helix, 68% of the coil, and 26% of the beta turns were seen in the analysis of the secondary structure of proteins found in the chimeric vaccine. GalaxyRefine tool was used to perform the refinement of 3D protein structure. An ideal vaccine candidate has several characteristics that RAMPAGE (Ramachandran plot) illustrated well. Most of the residues were found in the preferred locations, with only a few in the outliers. This shows that model quality is acceptable. The next step is very important in the construction of vaccines that will give impactful achievements.

TLR5 was selected for docking studies in the construction of omicron vaccine. In this study, CD4 + was taken to refine the binding energies and many interaction residues. The predicted binding energy of complex was − 48.61 kcal/mol^42^. In our study, a potential tool has been identified for molecular docking which involves immune-informatics computation. ClusPro2.0^26^ is an online server used for molecular docking of proteins and it was used for the estimation of refined vaccine model interaction by ligand binding domain of immune receptor ACE2. Multiple models were provided by docking studies. After checking 29 docked poses of the complex, top model was selected on the basis of active sites, having center energy − 1035.4 and lowest energy − 1215.1 kcal/mol. The docked complex with the lowest energy value showed more RMSD from the initial conformation.

The molecular dynamics simulation was run to check the dynamic behavior of the system. Figure 8 illustrates the findings in terms of numerous parameters. For example, it shows the complex structure and their MNA mobility, deformability values, B-factor, and elasticity of the complicated structure. There are decreased risks of deformation during an immune response when an optimally built complex is used. Covariance matrix analysis revealed immunological simulation of the vaccine construct, and the results were in line with the immune responses.

A similar study has applied immune-informatics approaches with simulation and docking for vaccine construct against TLR2 and TLR4 complexes. The current study proposed V1 Multi Epitope Vaccine (MEV) as a significant vaccine candidate that may help the scientific community to treat SARS-CoV-2 infections. In silico restriction cloning of the multi-epitope vaccine sequence into the pET-30a ( +) expression vector using SnapGene software^30^. Spike protein has boosted its binding affinity with ACE-2 receptors facilitating the entry of the omicron variant into the host cells. The developed vaccine was evaluated for immune-reactivity by expressing it in *E. coli* in order to validate it. The codon was tweaked to have a CAI of 1 and a GC content of 50.52% to have the best possible expression. *E. coli* GC concentration ranges from 30 to 70%, which is regarded as optimal for steady vector expression in *E. coli.* Using SnapGene software^30^, the optimized sequence was cloned into the pET-28a ( +) vector which created a new plasmid following in silico PCR, making it a recombinant plasmid. Several potential candidates for the vaccine have been considered with the help of in silico techniques. Compared to our chimeric vaccine, certain vaccine candidates have limited population coverage. As a result, in vitro and in vivo investigations demonstrating the efficacy of this vaccine candidate will be needed.

## Conclusion

In silico vaccine design with good population coverage and immune response offers a great opportunity for the identification of a possible candidate for clinical trials. In the present study, several computational approaches were used to construct effective vaccine against spike protein of omicron. B- and T-cell epitope were predicted using immuno-informatics approaches. Molecular docking using ClusPro shows a higher binding energy of − 1215.1 kcal/mol. Ramachandran plot shows 93% favored region. SnapGene tool showed good protein expression for our construct. In various in silico trials, our vaccine showed good immune response. Therefore, to establish the result of this study, further in vitro and in vivo experiments are required. The proposed vaccine met all the required criteria together with antigenicity, allergenicity, toxicity and other physiochemical properties. Collectively, our designed vaccine construct is safe, however, preclinical studies/validation must be performed before clinical trials.

## Supplementary Information

Supplementary Information 1.

Supplementary Information 2.

## Data Availability

All datasets on which the conclusions of the manuscript rely are presented in the paper. The raw data is available from corresponding authors (S.Z. and I.K.) on reasonable request.
