# Author Correction: Bioinformatics approach for the construction of multiple epitope vaccine against omicron variant of SARS-CoV-2

**DOI:** 10.1038/s41598-025-04451-0

**Published:** 2025-06-11

**Authors:** Sumera Zaib, Fatima Akram, Syed Talha Liaqat, Muhammad Zain Altaf, Imtiaz Khan, Ayed A. Dera, Jalal Uddin, Ajmal Khan, Ahmed Al-Harrasi

**Affiliations:** 1 Department of Basic and Applied Chemistry, Faculty of Science and Technology, University of Central Punjab, Lahore, 54590 Pakistan; 2 Department of Chemistry and Manchester Institute of Biotechnology, The University of Manchester, 131 Princess Street, Manchester, M1 7DN UK; 3 Department of Clinical Laboratory Sciences, College of Applied Medical Sciences, King Khalid University, Abha, Saudi Arabia; 4 Department of Pharmaceutical Chemistry, College of Pharmacy, King Khalid University, Abha, 62529 Kingdom of Saudi Arabia; 5 Natural and Medical Sciences Research Center, University of Nizwa, 616 Nizwa, Oman

Correction to: *Scientific Reports* 10.1038/s41598-022-23550-w, published online 09 November 2022

The original version of this Article contained errors in the Methodology and Results sections.

In the Methodology section, under the subheading ‘Vaccine construction’,

“An adjuvant 50S ribosomal protein L3 (UniProtKB—P60438) (https://www.uniprot.org/) was used on N terminal of vaccine. EAAAK, CPGPG, and AAY are the types of linker used to construct vaccine sequence^21^.”

now reads:

“An adjuvant 50S ribosomal protein L3 (UniProtKB—P60438) (https://www.uniprot.org/) was used on N terminal of vaccine. EAAAK, GPGPG, and AAY are the types of linker used to construct vaccine sequence^21^.”

Under the subheading ‘Assemblage of multi-epitope vaccine candidate sequence’,

“To form a sequence of vaccine, B- and T-cell epitopes, adjuvants, CPGPG, AAY and EAAAK were arranged manually.”

now reads:

“To form a sequence of vaccine, B- and T-cell epitopes, adjuvants, GPGPG, AAY and EAAAK were arranged manually.”

In the Results section, under the subheading ‘Vaccine construction’,

“Furthermore, T-cells and linkage of B-cells was made possible with the help of CPGPG and AYY linker, as shown in Fig. S2.”

now reads:

“Furthermore, T-cells and linkage of B-cells was made possible with the help of GPGPG and AAY linker, as shown in Fig. S2.”

The original Article has been corrected.

