# Supplementary material for: Bioinformatics approach for the construction of multiple epitope vaccine against omicron variant of SARS-CoV-2
Source: Sci Rep. 2022 Nov 9;12:19087. doi: 10.1038/s41598-022-23550-w (PMC9645332; doi:10.1038/s41598-022-23550-w)
Supplement: Supplementary file 1 — Supplementary Information 1. [file 41598_2022_23550_MOESM1_ESM.docx]

**Bioinformatics Approach for the Construction of Multiple Epitope Vaccine against Omicron Variant of SARS-CoV-2**

Sumera Zaib^1^*, Fatima Akram^1^, Syed Talha Liaqat^1^, Muhammad Zain Altaf^1^, Imtiaz Khan^2^*, Ayed A. Dera^3^, Jalal Uddin^4^, Ajmal Khan^5^, Ahmed Al-Harrasi^5^*

*^1^Department of Biochemistry, Faculty of Life Sciences, University of Central Punjab, Lahore-54590, Pakistan*

*^2^Department of Chemistry and Manchester Institute of Biotechnology, The University of Manchester, 131 Princess Street, Manchester M1 7DN, United Kingdom*

*^3^Department of Clinical Laboratory Sciences, College of Applied Medical Sciences, King Khalid University, Abha, Saudi Arabia*

*^4^Department of Pharmaceutical Chemistry, College of Pharmacy, King Khalid University, Abha 62529, Kingdom of Saudi Arabia*

*^5^Natural and Medical Sciences Research Center, University of Nizwa, Nizwa 616, Oman*

**Correspondence:** [sumera.zaib@ucp.edu.pk](mailto:sumera.zaib@ucp.edu.pk) (S.Z.); [kimtiaz@hotmail.co.uk](mailto:kimtiaz@hotmail.co.uk) (I.K.); [aharrasi@unizwa.edu.om](mailto:aharrasi@unizwa.edu.om) (A.A.-H)

**
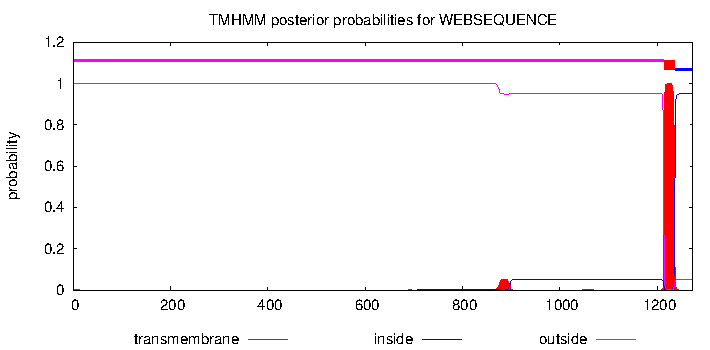
**

**Figure S1.** Prediction of transmembrane helices in proteins by TMHMM.

**Figure S2.** Vaccine construction using T- and B-cell epitopes and linkers (EAAAK, AAY, CPGPG).


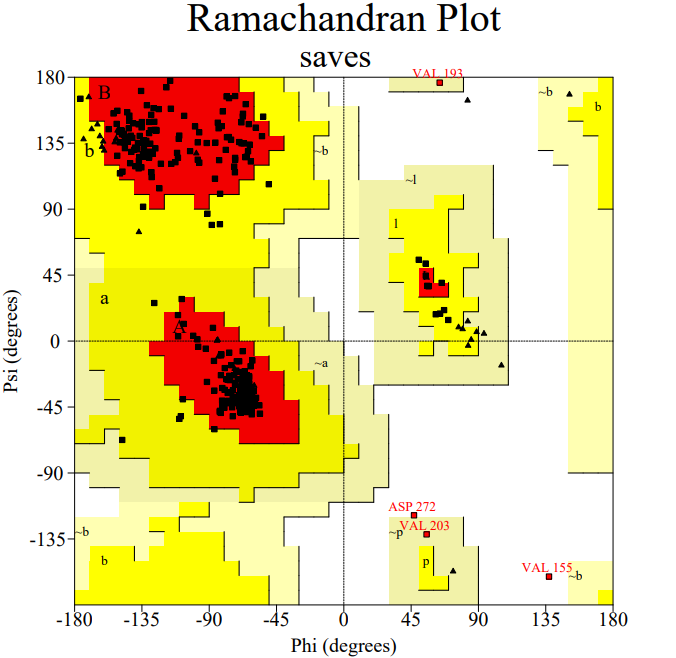


**Figure** **S3**. Refined tertiary structure validation by RAMPAGE server.

| **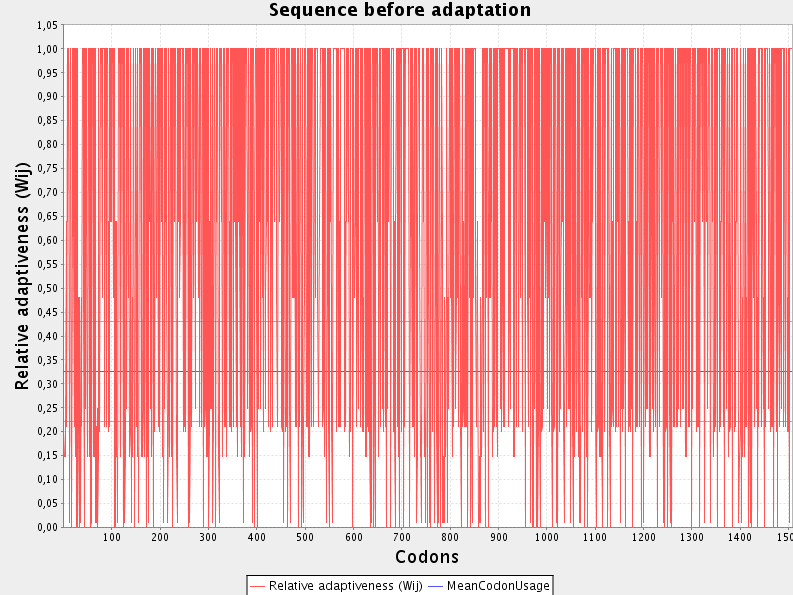**  **(a)** |
| --- |
| **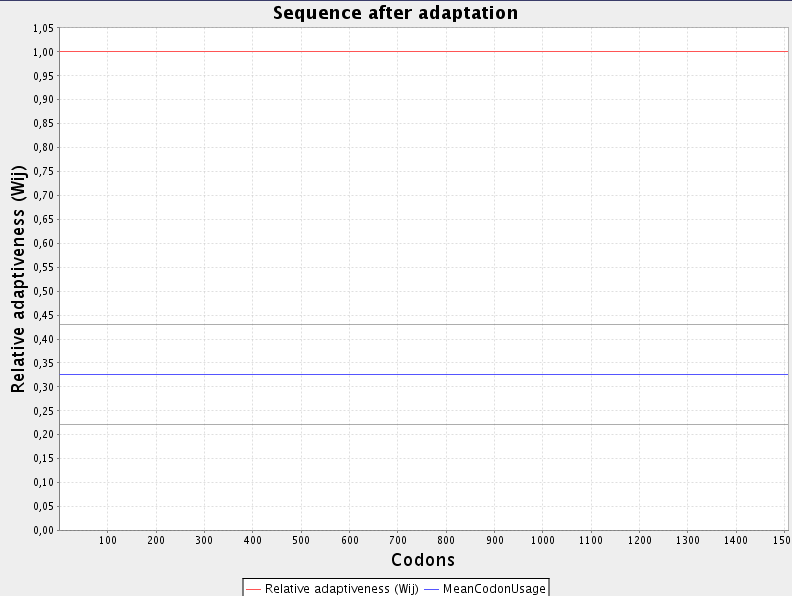**  **(b)** |
| **Figure S4**. Codon optimization of designed vaccine construct (**a**) sequence before adaptation; (**b**) *In silico* PCR amplification of vaccine construct followed by addition of restriction sites and cloning in pET-28a (+) vector. |
