# Supplementary figures and images for: Bioinformatics approach for the construction of multiple epitope vaccine against omicron variant of SARS-CoV-2
Source: Sci Rep. 2022 Nov 9;12:19087. doi: 10.1038/s41598-022-23550-w (PMC9645332; doi:10.1038/s41598-022-23550-w)

**TOC**


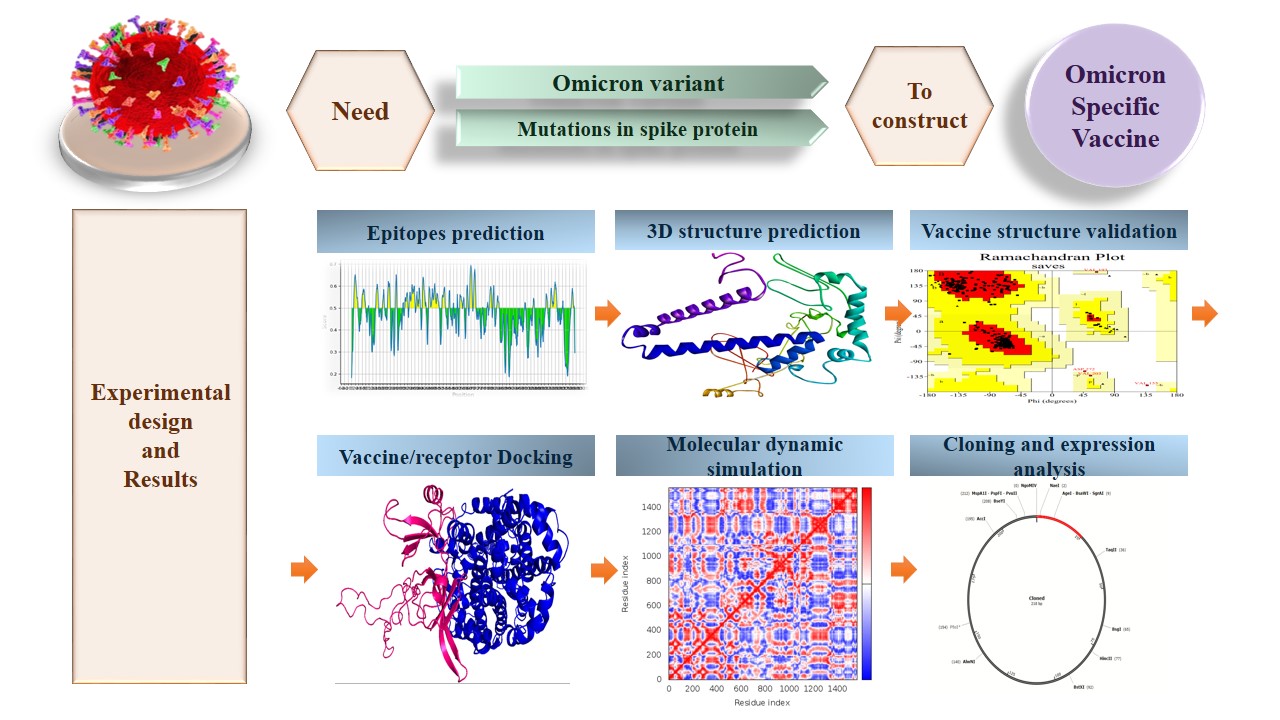

Supplement: Supplementary file 2 — Supplementary Information 2. [file 41598_2022_23550_MOESM2_ESM.docx]
